# High-Throughput SNP Genotyping

**DOI:** 10.1002/cfg.130

**Published:** 2002-02

**Authors:** Suzanne Jenkins, Neil Gibson

**Affiliations:** R&D Genetics AstraZeneca Mereside Alderley Park, Macclesfield Cheshire SK10 4TG UK

## Abstract

Whole genome approaches using single nucleotide polymorphism (SNP) markers have the potential to transform complex disease genetics and expedite pharmacogenetics research. This has led to a requirement for high-throughput SNP genotyping platforms. Development of a successful high-throughput genotyping platform depends on coupling reliable assay chemistry with an appropriate detection system to maximise efficiency with respect to accuracy, speed and cost. Current technology platforms are able to deliver throughputs in excess of 100 000 genotypes per day, with an accuracy of >99%, at a cost of 20–30 cents per genotype. In order to meet the demands of the coming years, however, genotyping platforms need to deliver throughputs in the order of one million genotypes per day at a cost of only a few cents per genotype. In addition, DNA template requirements must be minimised such that hundreds of thousands of SNPs can be interrogated using a relatively small amount of genomic DNA. As such, it is predicted that the next generation of high-throughput genotyping platforms will exploit large-scale multiplex reactions and solid phase assay detection systems.


**Comparative and Functional Genomics**
*Comp Funct Genom* 2002; **3**: 57–66.
**DOI:** 10.1002/cfg.130

**Review**

### High-throughput SNP genotyping

Suzanne Jenkins* and Neil Gibson
*R&D Genetics, AstraZeneca, Mereside, Alderley Park, Macclesfield, Cheshire, SK10 4TG, UK*

**Correspondence to:*
*Suzanne Jenkins, R&D Genetics, AstraZeneca, Mereside, Alderley Park, Macclesfield, Cheshire, SK10 4TG, UK.*
*E-mail: suzanne.jenkins@astrazeneca.com*

Received: 12 November 2001
Accepted: 19 November 2001
Published online: 5 December 2001

### Abstract

**Whole genome approaches using single nucleotide polymorphism (SNP) markers have the potential to transform complex disease genetics and expedite pharmacogenetics research. This has led to a requirement for high-throughput SNP genotyping platforms. Development of a successful high-throughput genotyping platform depends on coupling reliable assay chemistry with an appropriate detection system to maximise efficiency with respect to accuracy, speed and cost. Current technology platforms are able to deliver throughputs in excess of 100 000 genotypes per day, with an accuracy of >99%, at a cost of 20–30 cents per genotype. In order to meet the demands of the coming years, however, genotyping platforms need to deliver throughputs in the order of one million genotypes per day at a cost of only a few cents per genotype. In addition, DNA template requirements must be minimised such that hundreds of thousands of SNPs can be interrogated using a relatively small amount of genomic DNA. As such, it is predicted that the next generation of high-throughput genotyping platforms will exploit large-scale multiplex reactions and solid phase assay detection systems.** **Copyright © 2001 John Wiley & Sons, Ltd.**

**Keywords:** **SNP; genotyping technology; genetic variation; sequence detection; SNP detection; allelic discrimination; high-throughput**

### Introduction

Single nucleotide polymorphisms (SNPs) have gained wide acceptance as genetic markers for use in linkage and association studies. The SNP Consortium (TSC) and others have mapped >1.4 million SNP markers and placed this information within the public domain [34]. It has been estimated that, with the advent of a linkage disequilibrium (LD) map, around 100 000 SNP markers will be needed to carry out a whole genome association (WGA) study [21]. WGA approaches, which involve large-scale SNP genotyping, are expected to transform complex disease genetics and realise the potential of pharmacogenetics. To achieve this, very high-throughput SNP genotyping platforms are required.

The pharmaceutical industry, biotechnology companies and academic groups alike, aspire to carry out an increasing volume of SNP genotyping without increasing the cost of genetics research. To meet this demand, the search is on to find a genotyping technology able to deliver accurate genotyping at very high throughput and at low cost per genotype. In addition, the desired platform should be simple, reliable and flexible with respect to assay design and development. Current technologies can deliver >99% accuracy, at a rate of >100 K genotypes per day, at a cost of around 20–30 cents per genotype. Whilst this is a vast improvement on what was possible one to two years ago, we now seek a platform able to deliver >99% accuracy, at a rate of >1 million genotypes per day, costing a few cents per genotype. At the same time we wish to minimise DNA usage to around 1ng per genotype, or less.

In this review we discuss: those chemistries most suitable for high-throughput SNP genotyping; detection systems for high-throughput analysis of assay products; commercial platforms most suited to the high-throughput arena; and future trends in high-throughout genotyping technologies.

In addition to chemistry and detection preferences, it is important to consider how the output data of the preferred platform(s) will be interpreted. To achieve high-throughput genotyping, allele

Copyright © 2001 John Wiley & Sons, Ltd.

calling must be automated, accurate and reliable. Many technology providers favour automated allele calling systems which score confidence in the automatic call, requiring user intervention only where the score is lower than a predetermined threshold. Sample tracking and data management are also important considerations for high-throughput genotyping laboratories making some form of Laboratory Information Management System (LIMS) an essential component of any genotyping facility.

### High-throughput assay chemistry

There are a host of chemistries available for allelic discrimination, some more suitable for high-throughput SNP genotyping than others. Broadly speaking, methods are based on one or more of the following molecular mechanisms: 1. allele specific hybridisation; 2. flap endonuclease discrimination; 3. primer extension; 4. allele specific digestion; or 5. oligonucleotide ligation. In addition to being accurate and reliable, the chemistry needs to be inexpensive, readily amenable to automation and simple with respect to assay design and development.

### 1. Allele specific hybridisation (ASH)

The 5′ nuclease or TaqMan assay [25], dynamic allele specific hybridisation (DASH) [15], molecular beacons [40] and the scorpions assay [44] are all examples of ASH SNP genotyping technologies with high-throughput potential. Of these, TaqMan (Applied Biosystems,[proof note: URL to go on the next line] **http://www.appliedbiosystems.com**) has recently been adapted for high-throughput application [32]. Allelic discrimination depends on two allele specific probes, labelled with a probe-specific fluorescent dye and a generic quencher that reduces fluorescence in the intact probe. During amplification of the sequence surrounding the SNP, probes complimentary to the DNA target are cleaved by the 5′exonuclease activity of *Taq* polymerase (Figure 1a). Spatial separation of the dye and quencher results in an increase in probe-specific fluorescence, detectable using a plate reader.

Recent advances in TaqMan chemistry include the launch of a 384-well version of the platform, the use of a dark (non-fluorescent) quencher, and minor groove binder probes which allow the probe length to be shorter for improved allelic discrimination. In addition, an assay design and manufacture service (Assays-by-Design) is now offered by Applied Biosystems to facilitate the development of successful assays.

### 2. Flap endonuclease discrimination

The Invader assay (Third Wave Technologies Inc, **http://www.twt.com**) involves nuclease cleavage of a signal probe when two overlapping oligonucleotides hybridise to a complimentary DNA target. A generic invader probe and an allele specific primary probe are simultaneously hybridised to the target sequence such that they overlap at the site of a SNP when the primary probe is complimentary to the target. The triplex structure that results is recognised and cleaved by a flap endonuclease, releasing a probe specific tail sequence or 'flap' (Figure 1b). The cleaved fragment may be labelled with a probe specific fluorescent dye, which fluoresces following probe cleavage due to spatial separation from a quencher. Alternatively, the flap may act as the invader probe in a secondary reaction to amplify fluorescent signal (Invader squared) [12].

Recent modifications to the published method have increased applicability of this technology for high-throughput genotyping. PCR amplicons can be used as the DNA template for Invader reactions to reduce the requirement for DNA [28]. Furthermore, large multiplex (100-plex) PCR reactions have been shown to further reduce DNA requirements, minimise the cost implications of using PCR as a template and increase throughput of this platform [31]. Recently, it has been demonstrated that Invader reactions can be carried out in solid phase using oligonucleotide-bound streptavidin-coated particles [45]. This may provide a very high-throughput genotyping platform that requires a very small amount of DNA per genotype.

### 3. Primer extension

Primer extension, also known as mini-sequencing, forms the basis of a number of methods for allelic discrimination. An extension primer is annealed 5′ to a SNP, either adjacent to but not including the SNP or several bases upstream of the SNP. The primer is then extended for one or several nucleotides to include the SNP site. In the case of single base extension (SBE), a primer is annealed adjacent to a SNP and extended to incorporate a

Copyright © 2001 John Wiley & Sons, Ltd. 

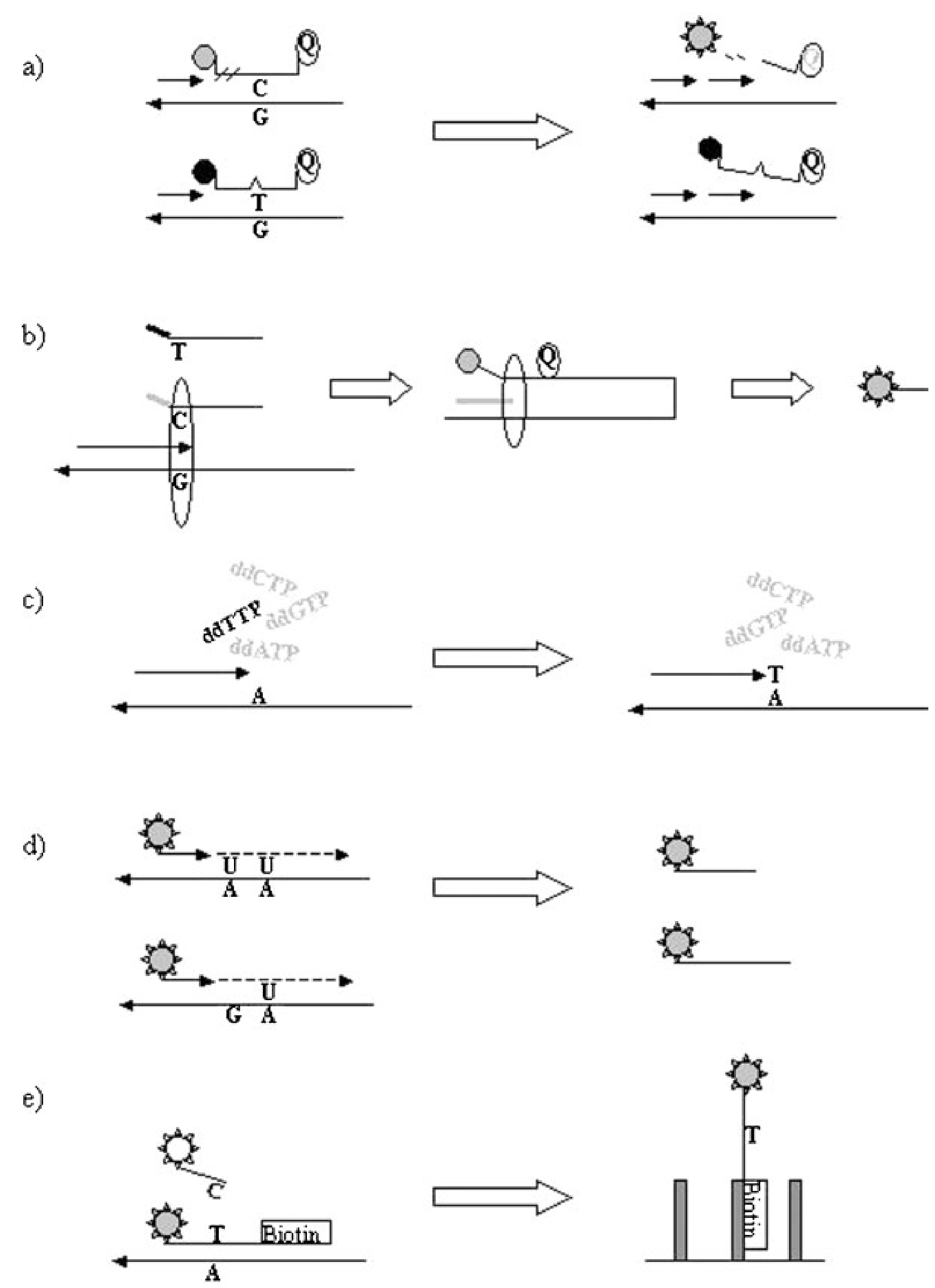

**Figure 1.** Allelic discrimination for a C/T SNP using five molecular mechanisms. a) TaqMan – the complimentary probe is cleaved during PCR to release probe-specific fluorescence. b) Invader squared – the triplex structure (circled), formed between the target sequence, invader probe and primary probe, is cleaved by a flap endonuclease. c) Single base extension – fluorescently labelled ddNTPs are incorporated into an extension primer in an allele dependent manner. d) SNaPIT – a diagnostic PCR primer is annealed close to the SNP and dUTP is incorporated in place of dTTP. UDG digestion results in different length fragments representing the different alleles. e) OLA – A fluorescently labelled allele-specific probe is hybridised to the target DNA and ligated to a generic biotin-labelled probe

Copyright © 2001 John Wiley & Sons, Ltd. 

dideoxynucleotide (ddNTP) at the polymorphic site (Figure 1c). SNaPshot (Applied Biosystems) involves differential fluorescent labelling of the four ddNTPs in a SBE reaction allowing fluorescent detection of the incorporated nucleotide. SNP-IT (Orchid Biosciences, **http://www.orchidbio.com**) is also based on fluorescent SBE and uses solid phase capture and detection of extension products. A further variant of primer extension is the Good assay [37],38] which involves extension of a primer modified near the 3′ end with a charged tag to increase sensitivity to mass spectrometry detection. Following extension with $\alpha$-S-(d)dNTPs, the unmodified 5′ end of the primer is digested leaving a short charged fragment suitable for high-throughput mass spectrometry analysis [37].

Alternatives to SBE include Pyrosequencing [1], allele specific primer extension (ASPE) and the amplification refractory mutation system (ARMS). Pyrosequencing involves sequential addition of dNTPs to an extension reaction. Incorporation of a nucleotide triggers an enzymatic cascade that releases a light signal, detected by a CCD camera. Liquid handling and detection has been automated in 384-well format for high-throughput genotyping. Allele specific primer extension (ASPE) and the amplification refractory mutation system (ARMS) are based on the same principle, namely that under optimised conditions, a primer will only be extended if the 3′ nucleotide is complimentary to the target sequence. These methods have recently been coupled to high-throughput detection systems, for example the Masscode system for mass spectrometry analysis of allele specific amplicons [18] and capture of ASPE products onto beads for detection by flow cytometry [46].

### 4. Allele specific digestion

Restriction fragment length polymorphism (RFLP) assays have been used extensively in the past to genotype SNPs but are less amenable to automation than many other methods. However, SNaPIT, an alternative method of allele specific digestion of PCR products, uses a generic approach that has potential for high-throughput genotyping [42]. The SNP and surrounding sequence is amplified in the presence of a modified dNTP, for example dUTP, using a diagnostic primer close to the SNP in either the forward or reverse direction. Subsequent digestion of the PCR product with a DNA glycosylase, for example uracil-DNA-glycosylase (UDG), results in different size fragments corresponding to the different alleles [41] (Figure 1d). The diagnostic primer can either be fluorescently labelled to enable separation and detection of the fragments using a capillary sequencer or unlabelled for mass spectrometry analysis. Assays are designed to maximise multiplexing potential.

### 5. Oligonucleotide ligation

The oligonucleotide ligation assay (OLA) was first described for allelic discrimination by Landegren *et al.* in 1988 [22] and has since been modified to exploit a themostable DNA Ligase [3]; interogate PCR templates [29]; and utilise a dual-colour detection system [35]. OLA involves ligation of two oligonucleotides, hybridised to a DNA template, one of which is allele specific such that it will only form part of a ligated product if it is complimentary to the target sequence (Figure 1e). OLA gave rise to another technique, Padlock [30], which involves circularisation of allele specific probes and it is Padlock that forms the basis of the rolling circle amplification (RCA) high-throughput genotyping technology [10]. Following circularisation of an allele specific open circle probe (OCP), exponential amplification is achieved using two primers. One of the primers is specific to the OCP backbone and is fluorescently labelled to distinguish between the allele specific OCPs. In addition, the labelled primer contains a hairpin loop and a quencher such that fluorescence is suppressed until the primer forms a double stranded molecule with the circularised probe. This disrupts the hairpin loop, separating the fluorophore and quencher and results in a detectable increase in fluorescent signal.

### Detection systems

Fluorescence is the most widely applied detection method currently employed for high-throughput genotyping. This is due to the ready availability of suitable fluorescent dyes and the diversity of their application. The use of fluorescence has been teamed with a number of different detection systems including plate readers, capillary electrophoresis and DNA arrays. In addition to fluorescence detection, mass spectrometry and light detection represent novel application of established technology for high-throughput genotyping.

Copyright © 2001 John Wiley & Sons, Ltd. 

Table 1. Fluorescent plate readers suitable for genotyping assay detection

| Vendor | Model | Features |
|---|---|---|
| Applied Biosystems | Cytofluor | 96 & 384 well plates |
| Applied Biosystems | 7700 | Laser excitation, CCD detection, realtime PCR applications, 96 well |
| Applied Biosystems | 7900 | Laser excitation, CCD detection, realtime PCR application, 384 well |
| Stratagene | Mx4000 | White light source, realtime PCR, 96 wells |
| Hybaid | Chimera | White light source, realtime PCR, 96 wells |
| APB | FarCyte | Up to 1536 wells. FI, FP, FRET, TRF, Luminescence |
| ThermoLabsystems | Fluoroskan Ascent | 96 & 384 well plates |
| Tecan | Ultra | Up to 1536 wells. FI, FP, TRF, luminescence and absorbance. |
| Tecan | Polarion | Xenon flash lamp, up to 384 wells. FI, FP. |
| LJL | Analyst, Acquest | 96 & 384 well plates, FI, FP, TRF, TR-FRET, Luminescence and Epi-Absorbance. |
| CRI | Affinity | Up to 1536 wells, FI, FP, FRET, Luminescence |
| Bio-Tek | FL-600 | Up to 384 wells, FI, Epifluorescence, Luminescence, Absorbance |
| Wallac (Perkin Elmer) | Viktor | Up to 384 wells, FI, TRF, luminescence, absorbance |

FI = Fluorescence intensity, FP = fluorescence polarisation, FRET = Fluorescence Resonance Energy Transfer, TRF = Time Resolved Fluorescence, TR-FRET = Time Resolved Fluorescence Resonance Energy Transfer

### Plate readers

There are many fluorescent plate readers capable of detecting fluorescence in a 96- or 384-well format and some of these are detailed in Table 1. Most models use a white light source and narrow band-pass filters to select the excitation and emission wavelengths and enable semi-quantitative steady state fluorescence intensity readings to be made. This technology has been applied to genotyping with TaqMan [32], Invader [13],31],28] and Rolling Circle Amplification [33],10]. Plate processing times are sufficiently fast to enable one instrument to read up to thirty 384-well plates per hour (over 10 000 determinations). This means that a single plate reader can measure fluorescence at the reaction end-point for $>100\,000$ reactions per day. Several readers are available which combine a PCR block with a fluorescence detection system to enable the user to monitor real-time changes in fluorescence throughout the course of the PCR (see Table 1). These readers are primarily used for quantitative RT-PCR but have also been applied to genotyping, mainly in the clinical field [14]. However, real-time analysis is inevitably slower than end-point reading and therefore, unsuitable for high-throughput genotyping.

Fluorescence plate readers are also available which will allow measurement of additional fluorescence parameters including polarisation, lifetime and time resolved fluorescence and fluorescence resonance energy transfer (see Table 1). Fluorescence polarisation has been applied to genotyping using ARMS [11], Invader [16] and TaqMan [23].

### Capillary electrophoresis

DNA capillary electrophoresis is a high-resolution technique for the separation of DNA fragments based on their size dependant mobility when passing through a sieving matrix. Following separation, DNA fragments are analysed for fluorescent signal as well as fragment size. The development of 96 capillary electrophoresis sequencers has increased the potential of this detection technique for SNP genotyping.

Sanger sequencing is widely used in SNP discovery and validation but its use in SNP genotyping is limited by its low throughput and the relatively high cost per sample. The development of sequencing technologies in microfabricated channels may deliver significantly reduced reagent costs and increases throughputs by decreasing reaction volumes and reducing run times [19],24],4],26],27]. Other SNP genotyping methods such as SNaPshot, based on SBE, and SNaPIT, based on allele specific digestion, exploit fluorescent sequencing detection technology to achieve higher throughputs and lower costs than sequencing through sample pooling and the use of short run times [5],42].

### DNA arrays

Oligonucleotide arrays, bound to a solid support, have been proposed as the future detection platform for high-throughput genotyping [36]. Two distinct approaches have been adopted, involving ASH whereby the oligonucleotide directly probes the target and tag arrays that capture solution phase

Copyright © 2001 John Wiley & Sons, Ltd. 

reaction products via hybridisation to their anti-tag sequences.

The HuSNP GeneChip (Affymetrix) contains allele specific probes for nearly 1500 SNPs [43]. The regions surrounding the SNPs are amplified by multiplex PCR and the amplicons are probed with the ASH array. This method is efficient for genotyping large numbers of SNPs for one to many DNA samples. Perlegen (**http://www.perlegen.com**) have exploited this approach and produced arrays in collaboration with Affymetrix that will enable them to perform genome wide SNP discovery and genotyping at high-density. Luminex (**http://www.luminexcorp.com**) have developed a panel of one hundred bead sets with unique fluorescent labels, identifiable using a flow analyser. The bead sets can be derivitised with allele specific oligonucleotides to create a bead-based array for multiplex genotyping by ASH [2].

Tag arrays are generic assemblies of oligonucleotides that are used to sort or deconvolute mixtures of oligos by hybridisation to the anti-tag sequences [6]. Affymetrix have produced the GenFlex array with 2000 tag sequences that are used to analyse the products of multiplex primer extension reactions [9]. Bead arrays have also been derivitised to form tag arrays and their application has been demonstrated in multiplex primer extension [8], rolling circle [20] and OLA [17] genotyping assays. Illumina (**http://www.illumina.com**) have produced a tag array where beads are bound to the tips of a bundle of fiber optic probes.

### Mass spectrometry

Many genotyping techniques involve the allele specific incorporation of two alternative nucleotides into an oligonucleotide probe. Mass spectrometry can be used to determine which variant nucleotide has been incorporated by measuring the mass of the products and this approach has been applied primarily to genotyping by primer extension [7] using the MALDI-TOF (Matrix Assisted Laser Desorption/Ionization – Time of Flight Mass Spectrometry) approach. There are many vendors of mass spectrometers including Agilent, Applied Biosystems, Bruker, GenTech, Hitachi, JEOL, Kratos, LECO, Micromass, Shimadzu, ThermoFinnigan, Varian and Waters.

The polyanionic nature of oligonucleotides results in low signal to noise, particularly for longer ($>$40 mer) fragments. This has been addressed by specifically cleaving long probes by acidolysis of P3′–N5′ phosphoramidate bonds [39] and by a combined approach whereby the probe is digested to a very short fragment which has been derivatised to lower its charge to a single positive or negative charge [37].

### Light

Pyrosequencing involves hybridisation of a sequencing primer to a single stranded template and sequential addition of individual dNTPs using a dedicated instrument [1]. Incorporation of a dNTP into a primer releases pyrophosphate that triggers a luciferase-catalyzed reaction. The light produced is detected by a charge coupled device (CCD) camera and each light signal is proportional to the number of nucleotides incorporated (**http://www.pyrosequencing.com**). The Pyrosequencing platform exemplifies novel application of existing technologies to develop solutions for high-throughput genotyping.

### Commercial platforms most suited to the high-throughput arena

To achieve high-throughput genotyping, the challenge lies in pairing the right assay chemistry with the right detection system to maximise efficiency with respect to accuracy, speed and cost. Many commercially available platforms have achieved this very successfully and some of these are outlined in Table 2. Platforms currently able to deliver $>$100 000 genotypes per instrument per day use fluorescent plate readers for detection of SNP alleles. However, such detection systems are less likely than others, such as those based on DNA arrays, to provide the basis of signal detection for the next generation of genotyping platforms due to reagent cost and DNA consumption considerations. As illustrated in Table 2, no single genotyping chemistry is emerging as the method of choice, although an increasing number of platforms employ primer extension chemistries, due to the potential for multiplexing.

### Future trends in high-throughput genotyping technologies

The next generation of high-throughput genotyping platforms are likely to employ an extensive scale of optimised multiplex reactions ($\sim$100-plex)

Copyright © 2001 John Wiley & Sons, Ltd. 

Table 2. Commercial platforms for high throughput genotyping

| Platform | Chemistry | Detection | Technology provider | Capacity per day[1] | Main Advantage | Web site |
|---|---|---|---|---|---|---|
| TaqMan® | TaqMan® | Plate reader | Applied Biosystems | > 100K | Assay design and manufacture service<br>Simple workflow | **http://www.appliedbiosystems.com** |
| Invader® | Invader® | Plate reader | Third Wave Technologies | > 100K | Assay design and manufacture service<br>PCR not necessary | **http://www.twt.com** |
| SNiPer | Rolling Circle Amplification | Plate reader | Amersham Pharmacia Biotech | 50K | Fully automated, integrated platform<br>PCR not necessary | **http://www.apbiotech.com** |
| ABI PRISM® SNaPshot™ | SnaPshot™(SBE) | Capillary electrophoresis | Applied Biosystems | ~20K | Simple assay design<br>Multiplexing | **http://www.appliedbiosystems.com** |
| SNaPIT | SNaPIT | Capillary electrophoresis | HiberGen | ~20K | Simple assay design<br>Multiplexing | **http://www.hibergen.com** |
| SNPstream 25K | SNP-IT(SBE) | DNA Arrays | ORCHID Biosciences | 25K | Fully automated, integrated platform<br>Multiplexing | **http://www.orchidbio.com** |
| MassARRAY | MassEXTENT™SNaPIT | Mass Spec | SEQUENOM | 50K | Inexpensive reagents<br>Multiplexing | **http://www.sequenom.com** |
| Masscode™ System | ARMS -Masscode tags | Mass Spec | QIAGEN Genomics | >40K | Proprietary Masscode tags | **http://www.Qiagen.com** |
| GOOD | GOOD Assay | Mass Spec | | 50K | Simple workflow<br>No purification | **http://www.cng.fr** |
| Pyrosequencing Preferred Technology Program (PTP) | Pyrosequencing™ | CCD camera | Pyrosequencing AB | 50K | Sequence around SNP provides confidence in genotype call<br>Allele quantification | **http://www.pyrosequencing.com** |

[1]Throughput is based on current capacity of detection instrument – additional liquid and data handling automation may be required to attain stated thoughput.

Copyright © 2001 John Wiley & Sons, Ltd.

combined with high-density DNA arrays. Primer extension currently forms the basis of many multiplex genotyping approaches (Table 2) and other chemistries such as OLA have considerable multiplex potential. In addition, any genotyping chemistry that can be developed for solid phase multiplexing, as demonstrated for the Invader technology [45], may spur future genotyping platforms. High-density DNA chips for analysis of extensive multiplex reactions may be based on the Affymetrix HuSNP (ASH) or GenFlex (tag) chips; bead arrays such as the Luminex bead system; or miniaturised bead arrays bound to Illumina's fibre optic bundles. Solid phase multiplex approaches will not only increase throughputs and drive down costs but will also reduce the amount of DNA required per genotype, making whole genome association studies feasible with limited DNA resources.

A number of technology providers such as Caliper (**http://www.calipertech.com**) and ACLARA Biosciences (**http://www.aclara.com**) are exploiting the use of microchannels as a way to miniaturise assay chemistry and detection. Both the LabChip™ system (Caliper) and LabCard (ACLARA Biosciences) offer automated SNP interrogation using very small volumes in microchannel networks. Minaturisation of genotyping reactions offers a reduction in both reagent cost and DNA requirements, whilst the level of automation facilitates high throughput of reactions.

Many technology providers are now developing SNP panels for whole genome linkage and association. This will make it possible to buy off-the-shelf assay panels at reduced cost compared with custom synthesis. For some platforms, such as those involving ASH DNA chips, the SNP panel is fixed, while for others, the use of a genome wide panel may be an option, with flexibility for custom synthesis where needed. The development of genome wide SNP panels, particularly when LD patterns have been characterised across the genome, will expedite the application of SNP genotyping for genome wide linkage and association studies.

As an alternative to high-throughput genotyping, a number of approaches have emerged for rapid identification of SNP allele frequency variation in different populations, such as disease affected and unaffected populations. For example, the LYNX Megatype technology (**http://www.lynxgen.com**) claims to compare the genomes of two populations in a single experiment. The technology is based on isolation of DNA fragments containing SNPs which differ in allele frequency between populations. In addition, a number of SNP genotyping technologies, including Pyrosequencing, appear to be suitable for allele quantification in pooled DNA samples. It remains to be seen whether such technologies will offer the sensitivity needed to detect small differences in allele frequency ($<5\%$) between population specific pools of samples.

### Conclusion

Homogeneous chemistries coupled with fluorescent plate reader detection currently offer the simplest route to developing a high-throughput genotyping platform. However, expensive reagent costs and relatively high DNA template requirements mean that miniaturisation of such methods will be necessary to meet future demands. An alternative is provided by the mass spectrometry platforms where the key advantage is the use of mass determination alone for allelic discrimination, removing the need for expensive primer labelling. Multiplex SNP genotyping strategies based on DNA arrays offer considerable potential for the next generation of platforms. The use of ASH arrays with fixed panels of SNPs may be a popular option for genome wide studies while greater flexibility can be offered using tag arrays. The laboratory processes associated with array detection are readily amenable to automation for very high-throughput genotyping.

It is likely that a well-positioned genotyping laboratory will need to be equipped with more than one platform to meet the demands of the coming years. This is because the most efficient and cost effective technologies for very high-throughput genotyping using genome-wide panels of SNP markers may not necessarily be suitable for high-density SNP analysis of smaller linked or associated regions. It remains to be seen which genotyping technology platforms will expedite genetics research in the Pharmacogenetics and Pharmacogenomics era.

### References

1. Alderborn A, Kristofferson A, Hammerling U. 2000. Determination of single-nucleotide polymorphisms by real-time pyrophosphate DNA sequencing. *Genome Res* **10**:1249–1258.

Copyright © 2001 John Wiley & Sons, Ltd. 

2. Armstrong B, Stewart M, Mazumder A. 2000. Suspension arrays for high throughput, multiplexed single nucleotide polymorphism genotyping. *Cytometry* **40**:102–108.
3. Barany F. 1991. Genetic disease detection and DNA amplification using cloned thermostable ligase. *Proc Natl Acad Sci U S A* **88**:189–193.
4. Barta C, Ronai Z, Nemoda Z, *et al.* 2001. Analysis of dopamine D4 receptor gene polymorphism using microchip electrophoresis. *J Chromatogr* **924**:285–290.
5. Barta C, Ronai Z, Sasvari-Szekely M, *et al.* 2001. Rapid single nucleotide polymorphism analysis by primer extension and capillary electrophoresis using polyvinyl pyrrolidone matrix. *Electrophoresis* **22**:779–782.
6. Ben-Dor A, Karp R, Schwikowski B, Yakhini Z. 2000. Universal DNA tag systems: a combinatorial design scheme. *J Comput Biol* **7**:503–519.
7. Bray MS, Boerwinkle E, Doris PA. 2001. High-throughput multiplex SNP genotyping with MALDI-TOF mass spectrometry: practice, problems and promise. *Hum Mutat* **17**: 296–304.
8. Chen J, Iannone MA, Li M-S, *et al.* 2000. A microsphere-based assay for multiplexed single nucleotide polymorphism analysis using single base chain extension. *Genome Res* **10**: 549–557.
9. Fan JB, Chen X, Halushka MK, *et al.* 2000. Parallel genotyping of human SNPs using generic high-density oligonucleotide tag arrays. *Genome Res* **10**: 853–860.
10. Faruqi AF, Hosono S, Driscoll MD, *et al.* 2001. High-throughput genotyping of single nucleotide polymorphisms with rolling circle amplification. *BMC Genomics* **2**: 4.
11. Gibson NJ, Gillard HL, Whitcombe D, *et al.* 1997. A homogeneous method for genotyping with fluorescence polarization. *Clin Chem* **43**:1336–1341.
12. Hall JG, Eis PS, Law SM, *et al.* 2000. Sensitive detection of DNA polymorphisms by the serial invasive signal amplification reaction. *Proc Natl Acad Sci U S A* **97**: 8272–8277.
13. Hessner MJ, Friedman KD, Voelkerding KV, *et al.* 2001. Multisite study for genotyping of the factor II (prothrombin) G20210a mutation by the invader assay. *Clin Chem* **47**: 2048–2050.
14. Hiratsuka M, Agatsuma Y, Omori F, *et al.* 2000. High throughput detection of drug-metabolizing enzyme polymorphisms by allele-specific fluorogenic 5′ nuclease chain reaction assay. *Biol Pharm Bull* **23**: 1131–1135.
15. Howell MW, Jobs M, Brookes AJ, *et al.* 1999. Dynamic allele-specific hybridisation. A new method for scoring single nucleotide polymorphisms. *Nat Biotechnol* **17**: 87–88.
16. Hsu TM, Law SM, Duan S, Neri BP, Kwok PY. 2001. Genotyping single-nucleotide polymorphisms by the invader assay with dual-color fluorescence polarization detection. *Clin Chem* **47**:1373–1377.
17. Iannone MA, J. Taylor D, Chen J, *et al.* 2000. Multiplexed single nucleotide polymorphism genotyping by oligonucleotide ligation and flow cytometry. *Cytometry* **39**: 131–140.
18. Kokoris M, Dix K, Moynihan K, *et al.* 2000. High-throughput SNP genotyping with the Masscode system. *Mol Diagn* **5**: 329–340.
19. Koutny L, Schmalzing D, Salas-Solano O, *et al.* 2000. Eight hundred-base sequencing in a microfabricated electrophoretic device. *Anal Chem* **72**:3388–3391.
20. Ladner DP, Leamon JH, Hamann S, *et al.* 2001. Multiplex detection of hotspot mutations by rolling circle-enabled universal microarrays. *Lab Invest* **81**: 1079–1086.
21. Eric L. 2001. Application of SNP technologies in medicine: lessons learned and future challenges. *Genome Res* **11**: 927–929.
22. Landegren U, Kaiser R, Sanders J, Hood L. 1988. A ligase-mediated gene detection technique. *Science* **241**: 1077–1080.
23. Latif S, Bauer-Sardina I, Ranade K, Livak KJ, Kwok PY. 2001. Fluorescence polarization in homogeneous nucleic acid analysis II: 5′-nuclease assay. *Genome Res* **11**: 436–440.
24. Liu S, Ren H, Gao Q, *et al.* 2000. Automated parallel DNA sequencing on multiple channel microchips. *Proc Natl Acad Sci U S A* **97**: 5369–5374.
25. Livak KJ, Marmaro J, Todd, JA. 1995. Towards fully automated genome-wide polymorphism screening. *Nat Genet* **9**: 341–342.
26. Medintz IL, Paegel BM, Mathies RA. 2001. Microfabricated capillary array electrophoresis DNA analysis systems. *J Chromatogr* **924**: 265–270.
27. Medintz I, Wong WW, Berti L, *et al.* 2001. High-performance multiplex SNP analysis of three hemochromatosis-related mutations with capillary array electrophoresis microplates. *Genome Res* **11**: 413–421.
28. Mein CA, Barratt BJ, Dunn MG, *et al.* 2000. Evaluation of single nucleotide polymorphism typing with invader on PCR amplicons and its automation. *Genome Res* **10**: 330–343.
29. Nickerson DA, Kaiser R, Lappin S, Stewart J, Hood L, Landegren U. 1990. Automated DNA diagnostics using an ELISA-based oligonucleotide ligation assay. *Proc Natl Acad Sci U S A* **87**: 8923–8927.
30. Nilsson M, Malmgren H, Samiotaki M, Kwiatkowski M, Chowdhary BP, Landegren U. 1994. Padlock probes: circularizing oligonucleotides for localized DNA detection. *Science* **265**: 2085–2088.
31. Ohnishi Y, Tanaka T, Ozaki K, Yamada R, Suzuki H, Nakamura Y. 2001. A high-throughput SNP typing system for genome-wide association studies. *J Hum Genet* **46**: 471–477.
32. Ranade K, Chang MS, Ting CT, *et al.* 2001. High-throughput genotyping with single nucleotide polymorphisms. *Genome Res* **11**: 1262–1268.
33. Rosler A, Bailey L, Jones S, *et al.* 2001. Rolling circle amplification for scoring single nucleotide polymorphisms. *Nucleosides Nucleotides Nucleic Acids* **20**: 893–894.
34. Sachidanandam R, Weissman D, Schmidt SC, *et al.* 2001. A map of human genome sequence variation containing 1.42 million single nucleotide polymorphisms. *Nature* **409**: 928–933.
35. Samiotaki M, Kwiatkowski M, Parik J, Landegren U. 1994. Dual-color detection of DNA sequence variants by ligase-mediated analysis. *Genomics* **20**: 238–242.
36. Sapolsky RJ, Hsie L, Berno A, Ghandour G, Mittmann M, Fan JB. 1999. High-throughput polymorphism screening and genotyping with high-density oligonucleotide arrays. *Genet Anal* **14**: 187–192.
37. Sauer S, Lechner D, Berlin K, *et al.* 2000. A novel procedure for efficient genotyping of single nucleotide polymorphisms. *Nucleic Acids Res* **28**: 13.
38. Sauer S, Lechner D, Berlin K, *et al.* 2000. Full flexibility genotyping of single nucleotide polymorphisms by the GOOD assay. *Nucleic Acids Res* **28**: 100.

Copyright © 2001 John Wiley & Sons, Ltd.

39. Shchepinov MS, Denissenko MF, Smylie KJ, *et al.* 2001. Matrix-induced fragmentation of P3′-N5′ phosphoramidate-containing DNA: high-throughput MALDI-TOF analysis of genomic sequence polymorphisms. *Nucleic Acids Res* **29**: 3864–3872.
40. Tyagi S, Kramer FR. 1996. Molecular beacons: probes that fluoresce upon hybridization. *Nat Biotechnol* **14**: 303–308.
41. Vaughan P. 2000. Use of uracil DNA glycosylase in the detection of known DNA mutations and polymorphisms. Glycosylase-mediated polymorphism detection (GMPD-check). *Methods Mol Bio* **152**: 169–177.
42. Vaughan P, McCarthy T. 1998. A novel process for mutation detection using uracil DNA-glycosylase. *Nucleic Acids Res* **26**: 810–815.
43. Wang DG, Fan J-B, Siao C-J, *et al.* 1998. Large-scale identification, mapping, and genotyping of single-nucleotide polymorphisms in the human genome. *Science* **280**: 1077–1082.
44. Whitcombe D, Theaker J, Guy SP, Brown T, Little S. 1999. Detection of PCR products using self-probing amplicons and fluorescence. *Nat Biotechnol* **17**: 804–807.
45. Wilkins-Stevens P, Hall JG, Lyamichev V, *et al.* 2001. Analysis of single nucleotide polymorphisms with solid phase invasive cleavage reactions. *Nucleic Acids Res* **29**: 77.
46. Ye F, Li MS, Taylor JD, *et al.* 2001. Fluorescent microsphere-based readout technology for multiplexed human single nucleotide polymorphism analysis and bacterial identification. *Hum Mutat* **17**: 305–316.

Copyright © 2001 John Wiley & Sons, Ltd. 

## References

[PDF_00624] Alderborn A., Kristofferson A., Hammerling U. (2000). Determination of single-nucleotide polymorphisms by real-time pyrophosphate DNA sequencing.. Genome Res.

[PDF_00630] Armstrong B., Stewart M., Mazumder A. (2000). Suspension arrays for high throughput, multiplexed single nucleotide polymorphism genotyping.. Cytometry.

[PDF_00633] Barany F. (1991). Genetic disease detection and DNA amplification using cloned thermostable ligase.. Proc Natl Acad Sci U S A.

[PDF_00636] Barta C., Ronai Z., Nemoda Z., Szekely A., Kovacs E., Sasvari-Szekely M., Guttman A. (2001). Analysis of dopamine D4 receptor gene polymorphism using microchip electrophoresis.. J Chromatogr A.

[PDF_00639] Barta C., Ronai Z., Sasvari-Szekely M., Guttman A. (2001). Rapid single nucleotide polymorphism analysis by primer extension and capillary electrophoresis using polyvinyl pyrrolidone matrix.. Electrophoresis.

[PDF_00643] Ben-Dor A., Karp R., Schwikowski B., Yakhini Z. (2000). Universal DNA tag systems: a combinatorial design scheme.. J Comput Biol.

[PDF_00646] Bray M. S., Boerwinkle E., Doris P. A. (2001). High-throughput multiplex SNP genotyping with MALDI-TOF mass spectrometry: practice, problems and promise.. Hum Mutat.

[PDF_00650] Chen J., Iannone M. A., Li M. S., Taylor J. D., Rivers P., Nelsen A. J., Slentz-Kesler K. A., Roses A., Weiner M. P. (2000). A microsphere-based assay for multiplexed single nucleotide polymorphism analysis using single base chain extension.. Genome Res.

[PDF_00654] Fan J. B., Chen X., Halushka M. K., Berno A., Huang X., Ryder T., Lipshutz R. J., Lockhart D. J., Chakravarti A. (2000). Parallel genotyping of human SNPs using generic high-density oligonucleotide tag arrays.. Genome Res.

[PDF_00660] Gibson N. J., Gillard H. L., Whitcombe D., Ferrie R. M., Newton C. R., Little S. (1997). A homogeneous method for genotyping with fluorescence polarization.. Clin Chem.

[PDF_00663] Hall J. G., Eis P. S., Law S. M., Reynaldo L. P., Prudent J. R., Marshall D. J., Allawi H. T., Mast A. L., Dahlberg J. E., Kwiatkowski R. W. (2000). Sensitive detection of DNA polymorphisms by the serial invasive signal amplification reaction.. Proc Natl Acad Sci U S A.

[PDF_00666] Hessner M. J., Friedman K. D., Voelkerding K. V., Huber S., Ryan D., Nuccie B., Ledford M. (2001). Multisite study for genotyping of the factor II (prothrombin) G20210A mutation by the invader assay.. Clin Chem.

[PDF_00670] Hiratsuka M., Agatsuma Y., Omori F., Narahara K., Inoue T., Kishikawa Y., Mizugaki M. (2000). High throughput detection of drug-metabolizing enzyme polymorphisms by allele-specific fluorogenic 5' nuclease chain reaction assay.. Biol Pharm Bull.

[PDF_00674] Howell W. M., Jobs M., Gyllensten U., Brookes A. J. (1999). Dynamic allele-specific hybridization. A new method for scoring single nucleotide polymorphisms.. Nat Biotechnol.

[PDF_00677] Hsu T. M., Law S. M., Duan S., Neri B. P., Kwok P. Y. (2001). Genotyping single-nucleotide polymorphisms by the invader assay with dual-color fluorescence polarization detection.. Clin Chem.

[PDF_00681] Iannone M. A., Taylor J. D., Chen J., Li M. S., Rivers P., Slentz-Kesler K. A., Weiner M. P. (2000). Multiplexed single nucleotide polymorphism genotyping by oligonucleotide ligation and flow cytometry.. Cytometry.

[PDF_00684] Kokoris M., Dix K., Moynihan K., Mathis J., Erwin B., Grass P., Hines B., Duesterhoeft A. (2000). High-throughput SNP genotyping with the Masscode system.. Mol Diagn.

[PDF_00687] Koutny L., Schmalzing D., Salas-Solano O., El-Difrawy S., Adourian A., Buonocore S., Abbey K., McEwan P., Matsudaira P., Ehrlich D. (2000). Eight hundred-base sequencing in a microfabricated electrophoretic device.. Anal Chem.

[PDF_00690] Ladner D. P., Leamon J. H., Hamann S., Tarafa G., Strugnell T., Dillon D., Lizardi P., Costa J. (2001). Multiplex detection of hotspot mutations by rolling circle-enabled universal microarrays.. Lab Invest.

[PDF_00696] Landegren U., Kaiser R., Sanders J., Hood L. (1988). A ligase-mediated gene detection technique.. Science.

[PDF_00698] Latif S., Bauer-Sardina I., Ranade K., Livak K. J., Kwok P. Y. (2001). Fluorescence polarization in homogeneous nucleic acid analysis II: 5'-nuclease assay.. Genome Res.

[PDF_00701] Liu S., Ren H., Gao Q., Roach D. J., Loder R. T., Armstrong T. M., Mao Q., Blaga I., Barker D. L., Jovanovich S. B. (2000). Automated parallel DNA sequencing on multiple channel microchips.. Proc Natl Acad Sci U S A.

[PDF_00704] Livak K. J., Marmaro J., Todd J. A. (1995). Towards fully automated genome-wide polymorphism screening.. Nat Genet.

[PDF_00707] Medintz I. L., Paegel B. M., Mathies R. A. (2001). Microfabricated capillary array electrophoresis DNA analysis systems.. J Chromatogr A.

[PDF_00710] Medintz I., Wong W. W., Berti L., Shiow L., Tom J., Scherer J., Sensabaugh G., Mathies R. A. (2001). High-performance multiplex SNP analysis of three hemochromatosis-related mutations with capillary array electrophoresis microplates.. Genome Res.

[PDF_00714] Mein C. A., Barratt B. J., Dunn M. G., Siegmund T., Smith A. N., Esposito L., Nutland S., Stevens H. E., Wilson A. J., Phillips M. S. (2000). Evaluation of single nucleotide polymorphism typing with invader on PCR amplicons and its automation.. Genome Res.

[PDF_00718] Nickerson D. A., Kaiser R., Lappin S., Stewart J., Hood L., Landegren U. (1990). Automated DNA diagnostics using an ELISA-based oligonucleotide ligation assay.. Proc Natl Acad Sci U S A.

[PDF_00722] Nilsson M., Malmgren H., Samiotaki M., Kwiatkowski M., Chowdhary B. P., Landegren U. (1994). Padlock probes: circularizing oligonucleotides for localized DNA detection.. Science.

[PDF_00726] Ohnishi Y., Tanaka T., Ozaki K., Yamada R., Suzuki H., Nakamura Y. (2001). A high-throughput SNP typing system for genome-wide association studies.. J Hum Genet.

[PDF_00729] Ranade K., Chang M. S., Ting C. T., Pei D., Hsiao C. F., Olivier M., Pesich R., Hebert J., Chen Y. D., Dzau V. J. (2001). High-throughput genotyping with single nucleotide polymorphisms.. Genome Res.

[PDF_00732] Rösler A., Bailey L., Jones S., Briggs J., Cuss S., Horsey I., Kenrick M., Kingsmore S., Kent L., Pickering J. (2001). Rolling circle amplification for scoring single nucleotide polymorphisms.. Nucleosides Nucleotides Nucleic Acids.

[PDF_00735] Sachidanandam R., Weissman D., Schmidt S. C., Kakol J. M., Stein L. D., Marth G., Sherry S., Mullikin J. C., Mortimore B. J., Willey D. L. (2001). A map of human genome sequence variation containing 1.42 million single nucleotide polymorphisms.. Nature.

[PDF_00739] Samiotaki M., Kwiatkowski M., Parik J., Landegren U. (1994). Dual-color detection of DNA sequence variants by ligase-mediated analysis.. Genomics.

[PDF_00743] Sapolsky R. J., Hsie L., Berno A., Ghandour G., Mittmann M., Fan J. B. (1999). High-throughput polymorphism screening and genotyping with high-density oligonucleotide arrays.. Genet Anal.

[PDF_00754] Shchepinov M. S., Denissenko M. F., Smylie K. J., Wörl R. J., Leppin A. L., Cantor C. R., Rodi C. P. (2001). Matrix-induced fragmentation of P3'-N5' phosphoramidate-containing DNA: high-throughput MALDI-TOF analysis of genomic sequence polymorphisms.. Nucleic Acids Res.

[PDF_00759] Tyagi S., Kramer F. R. (1996). Molecular beacons: probes that fluoresce upon hybridization.. Nat Biotechnol.

[PDF_00765] Vaughan P., McCarthy T. V. (1998). A novel process for mutation detection using uracil DNA-glycosylase.. Nucleic Acids Res.

[PDF_00761] Vaughan P. (2000). Use of uracil DNA glycosylase in the detection of known DNA mutations and polymorphisms. Glycosylase-mediated polymorphism detection (GMPD-check).. Methods Mol Biol.

[PDF_00768] Wang D. G., Fan J. B., Siao C. J., Berno A., Young P., Sapolsky R., Ghandour G., Perkins N., Winchester E., Spencer J. (1998). Large-scale identification, mapping, and genotyping of single-nucleotide polymorphisms in the human genome.. Science.

[PDF_00772] Whitcombe D., Theaker J., Guy S. P., Brown T., Little S. (1999). Detection of PCR products using self-probing amplicons and fluorescence.. Nat Biotechnol.

[PDF_00778] Ye F., Li M. S., Taylor J. D., Nguyen Q., Colton H. M., Casey W. M., Wagner M., Weiner M. P., Chen J. (2001). Fluorescent microsphere-based readout technology for multiplexed human single nucleotide polymorphism analysis and bacterial identification.. Hum Mutat.

